# Study on External Gas-Assisted Mold Temperature Control with the Assistance of a Flow Focusing Device in the Injection Molding Process

**DOI:** 10.3390/ma14040965

**Published:** 2021-02-18

**Authors:** Nguyen Truong Giang, Pham Son Minh, Tran Anh Son, Tran Minh The Uyen, Thanh-Hai Nguyen, Hung-Son Dang

**Affiliations:** 1 Ho Chi Minh City University of Technology (HCMUT), 268 Ly Thuong Kiet Street, District 10, Ho Chi Minh City 72506, Vietnam; ntgiang.sdh19@hcmut.edu.vn (N.T.G.); haint@hcmut.edu.vn (T.-H.N.); 2 Vietnam National University Ho Chi Minh City, Linh Trung Ward, Thu Duc District 71300, Vietnam; 3 HCMC University of Technology and Education, Hochiminh City 71307, Vietnam; minhps@hcmute.edu.vn (P.S.M.); uyentmt@hcmute.edu.vn (T.M.T.U.); sondh@hcmute.edu.vn (H.-S.D.)

**Keywords:** injection molding, mold temperature control, external gas-assisted mold temperature control (Ex-GMTC), cavity temperature distribution, flow focusing device (FFD), melt flow length, heating rate, temperature uniformity

## Abstract

In the injection molding field, the flow of plastic material is one of the most important issues, especially regarding the ability of melted plastic to fill the thin walls of products. To improve the melt flow length, a high mold temperature was applied with pre-heating of the cavity surface. In this paper, we present our research on the injection molding process with pre-heating by external gas-assisted mold temperature control. After this, we observed an improvement in the melt flow length into thin-walled products due to the high mold temperature during the filling step. In addition, to develop the heating efficiency, a flow focusing device (FFD) was applied and verified. The simulations and experiments were carried out within an air temperature of 400 °C and heating time of 20 s to investigate a flow focusing device to assist with external gas-assisted mold temperature control (Ex-GMTC), with the application of various FFD types for the temperature distribution of the insert plate. The heating process was applied for a simple insert model with dimensions of 50 mm × 50 mm × 2 mm, in order to verify the influence of the FFD geometry on the heating result. After that, Ex-GMTC with the assistance of FFD was carried out for a mold-reading process, and the FFD influence was estimated by the mold heating result and the improvement of the melt flow length using acrylonitrile butadiene styrene (ABS). The results show that the air sprue gap (h) significantly affects the temperature of the insert and an air sprue gap of 3 mm gives the best heating rate, with the highest temperature being 321.2 °C. Likewise, the actual results show that the height of the flow focusing device (V) also influences the temperature of the insert plate and that a 5 mm high FFD gives the best results with a maximum temperature of 332.3 °C. Moreover, the heating efficiency when using FFD is always higher than without FFD. After examining the effect of FFD, its application was considered, in order to improve the melt flow length in injection molding, which increased from 38.6 to 170 mm, while the balance of the melt filling was also clearly improved.

## 1. Introduction

In today’s industries, with the development of a wide range of product geometries, injection molding has become known as one of the most popular plastic manufacturing processes because of its low-cost advantages and high efficiency. In general, an injection molding cycle includes three stages. In the first stage of the process, the liquid melt is created by heating the raw material in an injection molding machine. Then, the formation of the plastic product is carried out by pressing the hot melt into the cavity. At the final stage, after the cooling time, the mold reaches the ejection temperature and it is ready to open, meaning that the product can be removed. It can be seen that the hot melt filling step is very important for the shaping of the parts, as well as in relation to the quality of the product itself. This step depends on several elements, such as the material viscosity, melt temperature, melt flow thickness, injection pressure, injection speed, and mold temperature. In recent studies, for improving the melt flow ability and reducing the injection pressure, a molding cycle with a high mold temperature has been investigated. The results show that with a higher cavity temperature, the melt can flow into the narrow cavity more easily, but this leads to a longer cooling time. In contrast, with a lower cavity temperature, the cooling step is completed more rapidly, and it is difficult to fill the cavity with the hot melt because of the appearance of frozen layers [1]. Due to the higher demands of customers in today’s industries in terms of the product quality and complex product geometries, the mold temperature control method has been considered as one of the most effective technologies for solving the problems that occur during the filling step in the injection molding process [2,3]. For example, the mold temperature for manufacturing micro products or thin-walled products must be high in the filling step to meet its requirements, but a short cycle time must be maintained. Therefore, a variety of methods have been tested for increasing the mold temperature.

In the primary study, the steam heating method was implemented with a hot fluid flowing inside the mold and a cooling channel, in order to increase the mold temperature to the expected value. For an expected mold temperature under 100 °C, hot water was employed; however, when the mold temperature was higher than 100 °C, another fluid was used, such as hot oil, steam, or water under high pressure. In general, during the heating process, these methods require the addition of mold equipment and the high pressure of the fluid should be maintained throughout the operation time [4,5].

When the heating target is higher than 100 °C, the mold plate is often heated by electronic heaters [6]. This is a popular solution for raising the mold temperature; however, this method often causes the mold cycle to become longer. As a result, the use of many cooling channels was suggested for the cooling step. In previous research, after the heating process, a mold temperature of 150 °C could be reached and the results also indicate a significant reduction in the number of frozen layers [7,8]. However, this method also requires additional heating sources and tool costs for mold design.

Due to energy wasted when the heating step was applied for all mold plate volumes, mold surface heating methods were suggested, such as infrared heating, laser heating, induction heating, and gas heating. The results showed an improvement in the energy consumption and heating rate using an infrared system in the heating process. In this way, the center of the mold surface could reach over 180 °C after the heating process [9,10,11]. For microinjection molding, laser heating was considered for mold temperature control [12,13] because it is advantageous for small products with a high heating rate; however, this method required coating of the cavity surface and additional equipment. In addition, induction heating was suggested as a method that had a rapid heating rate with a proper coil design [14]. The induction heating method was verified with several heating methods, such as proximity heating [15,16], ring heating [17], and coil heating [18]. The heating process with the application of the induction heating method is easy to predict using a simulation; however, this method also remains disadvantageous due to the requirement for proper coil design and the potential for over-heating, as well as the fact that aluminum plates are hardly suitable for heating using this method.

In addition, and in relation to surface heating, gas-assisted mold temperature control (GMTC) was investigated [19,20,21]. In this heating method, the mold temperature reaches the target value due to the heat convection between the cavity surface and the hot gas that flows into the cavity. The results proved that the cavity surface could be increased to over 200 °C. However, the requirement for a complex mold structure design because of the heating system integrated into the mold is also a problem that needs to be resolved in further research. Then, because of the drawbacks of GMTC, external GMTC (Ex-GMTC) was suggested, with the hot gas sprue being directly applied to the cavity surface for heating before the start of the next molding cycle [22]. This development does possess some advantages, such as its higher heating rate and better temperature distribution for many cavity geometries, and the fact that easier local heating of the mold plate is possible. Ex-GMTC has also succeeded in improving the weld line quality [23] and melt flow length [24]. However, these studies also show that Ex-GMTC is strongly influenced by the environment, as air flows from another source, meaning that the hot gas can be easily dispersed and, consequently, the heating efficiency and heating stability are reduced.

According to these disadvantages, in this paper, a flow focusing device (FFD) was suggested for controlling the hot gas flow, in order to improve the heating rate and mold temperature. To achieve this, the FFD was applied to investigate the factors that affect the temperature distribution on the insert plate in various simulations and experiments during the gas heating process, such as the air sprue gap, the height of the FFD, and the number of outlet holes on the side and top surfaces of the air cover. In addition, an application of FFD to improve the melt flow length was also conducted. The heating process was achieved with four hot gas gates on a melt flow length mold. In this application, an FFD was designed and manufactured for application in four hot gas gates. After finishing the injection molding process, the melt flow length was measured to observe the influence of Ex-GMTC with the application of FFD on the melt flow length. In all cases, simulations were used to predict the heating rate and temperature distribution. Experimentally, the temperature distribution of the cavity surface was determined by the Fluke TiS20 infrared camera (Fluke Corporation, Everett, Washington, DC, USA). The heating process was carried out 10 times for each case. After this, the average temperature result was calculated for a discussion and comparison with the simulation result.

## 2. Simulation and Experimental Methods

### 2.1. Experimental Methods

**Figure 1:**
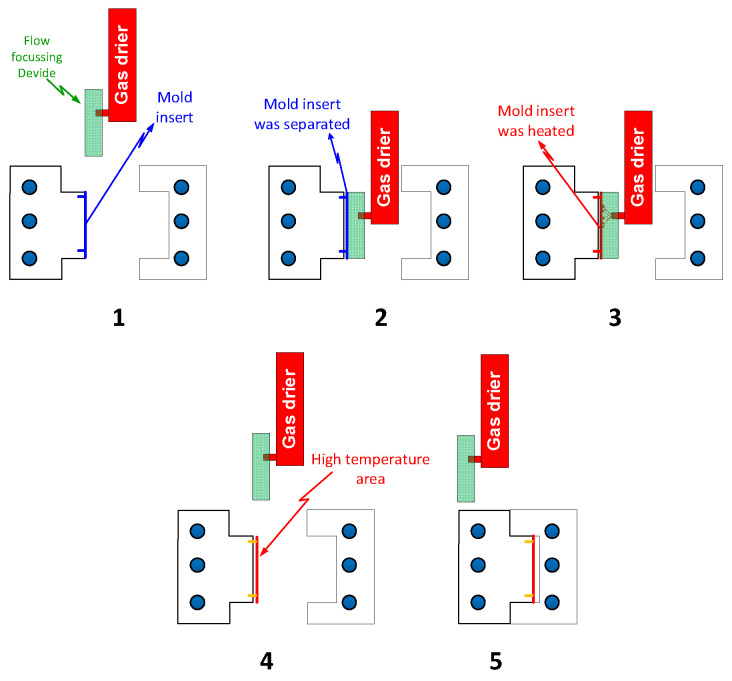
The heating steps of external gas-assisted mold temperature control with the assistance of a flow focusing device (FFD) in the injection molding process: Two-half molds are moved to the heating position (**1**); the hot gas source is moved to the heating area (**2**); the heating area is heated by the hot gas (**3**); the hot gas source is removed (**4**); and the two-half molds are closed for starting the molding cycle (**5**).

In this study, the Ex-GMTC method was applied in the injection molding process with five steps, as shown in Figure 1. In step 1, after the molding cycle is finished and the product is rejected, the two-half molds are moved to the heating position (step 1). Then, the hot gas source is moved to the heating area, as in step 2. In this step, the insert is separated from the mold plate to reduce the heat transferred from it to the mold plate. Next, the heating area is heated by the hot gas that sprues directly into the heating surface of the insert (step 3). Because of heat convection, the cavity surface receives thermal energy and its temperature will rise to the expected temperature. At the end of the heating process, the cavity is maintained at a high temperature. In step 4, when the heating area reaches the target temperature, the hot gas source is removed to the outside of the molding area. Then, the two-half molds close after the gas drier is completely removed from the heating area. In the closing period of the mold, the insert position is moved to the molding location, in contact with the mold plate (step 5). When the two-half molds are closed, the melt is pressed so that it fills the cavity, in order to begin a new molding cycle. In this step, due to the high temperature of the mold surface, the hot melt can easily flow into the cavity.

**Figure 2:**
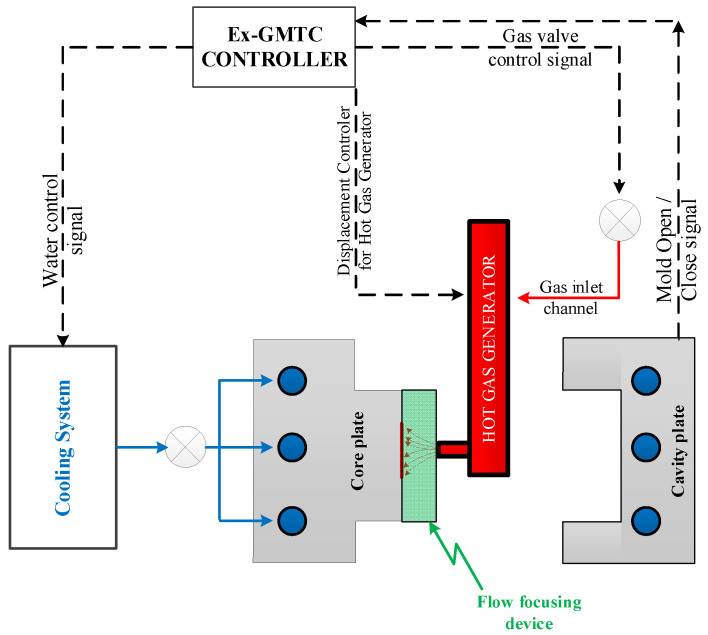
Schematic of the external gas-assisted mold temperature control (Ex-GMTC) system with the assistance of FFD.

Figure 2 illustrates the assembly of the heating system, which includes an Ex-GMTC controller, a hot gas with 12 kW of power, and a cooling system. The robot arm was controlled by the controller, in order to move the hot gas generator to the heating area. The outside dimensions of the air drier were 240 mm × 100 mm × 60 mm and this was used to produce hot air with an inlet air pressure of 0.7 MPa. Then, hot gas flowed out of the 10 mm diameter gas gate. The gas drier system supported a heat source with a maximum gas temperature of 400 °C and a maximum heating time of 60 s.

**Figure 3:**
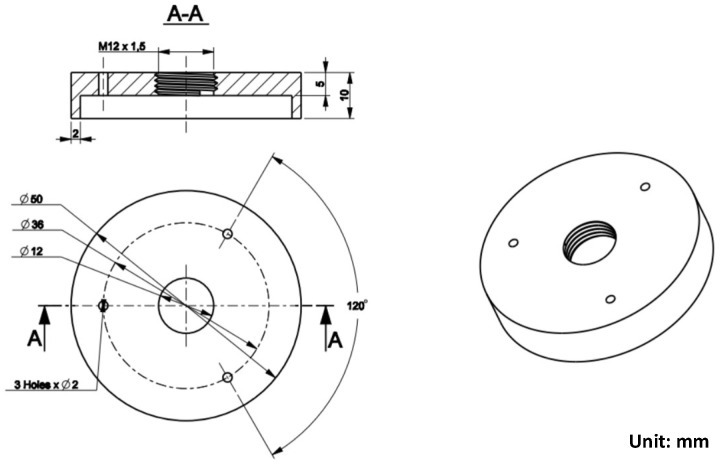
Design of the flow focusing device for hot air.

**Figure 4:**
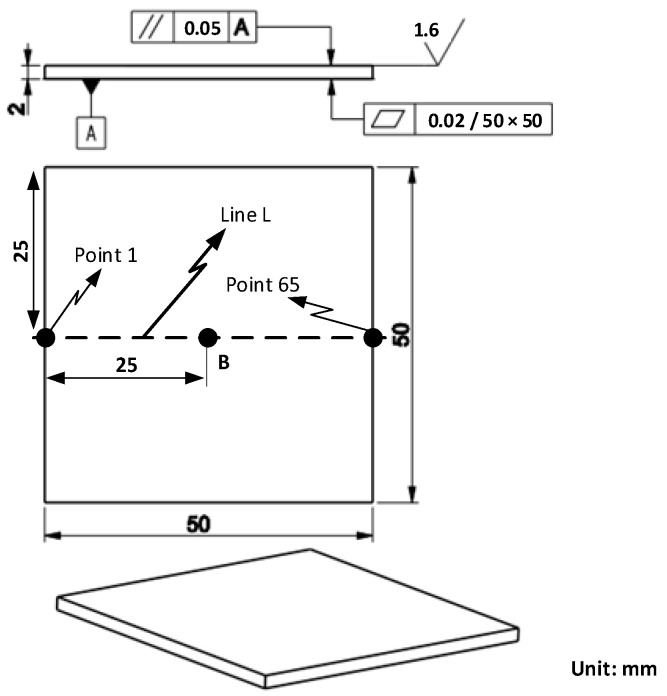
Design of insert plates.

**Figure 5:**
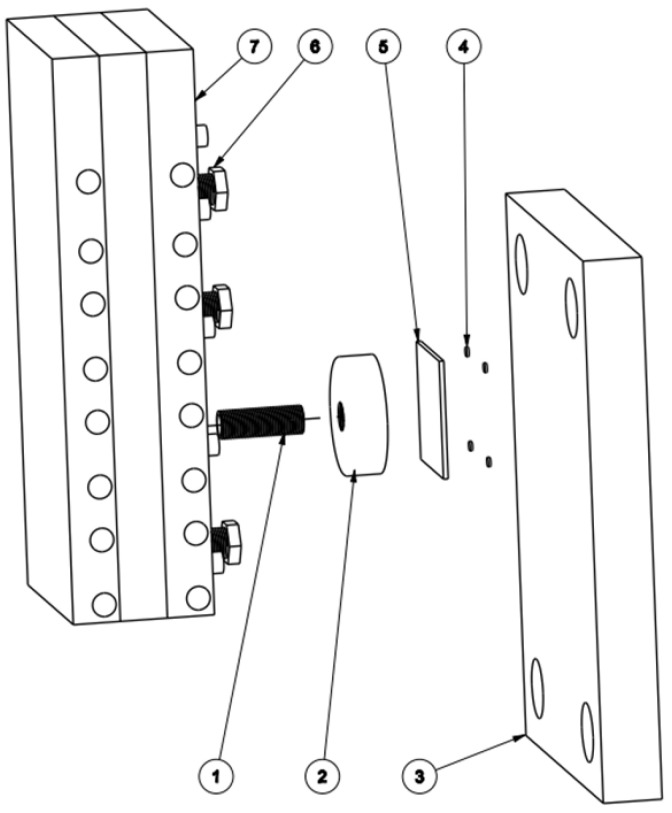
Heating process with air cover. (**1**) Threaded rod; (**2**) air cover; (**3**) core; (**4**) magnet; (**5**) insert plate; (**6**) screw M13; (**7**) heater.

In this research, for focusing the hot air flow into the heating area, a flow focusing device (FFD) was applied. The design of the FFD is presented in Figure 3. Its application in the heating process was observed to assess the temperature distribution on the insert plate, as shown in Figure 4. The insert plate had dimensions of 50 mm × 50 mm and a thickness of 2 mm. The FFD was connected to the hot gas generator and the insert, as shown in Figure 5.

**Figure 6:**
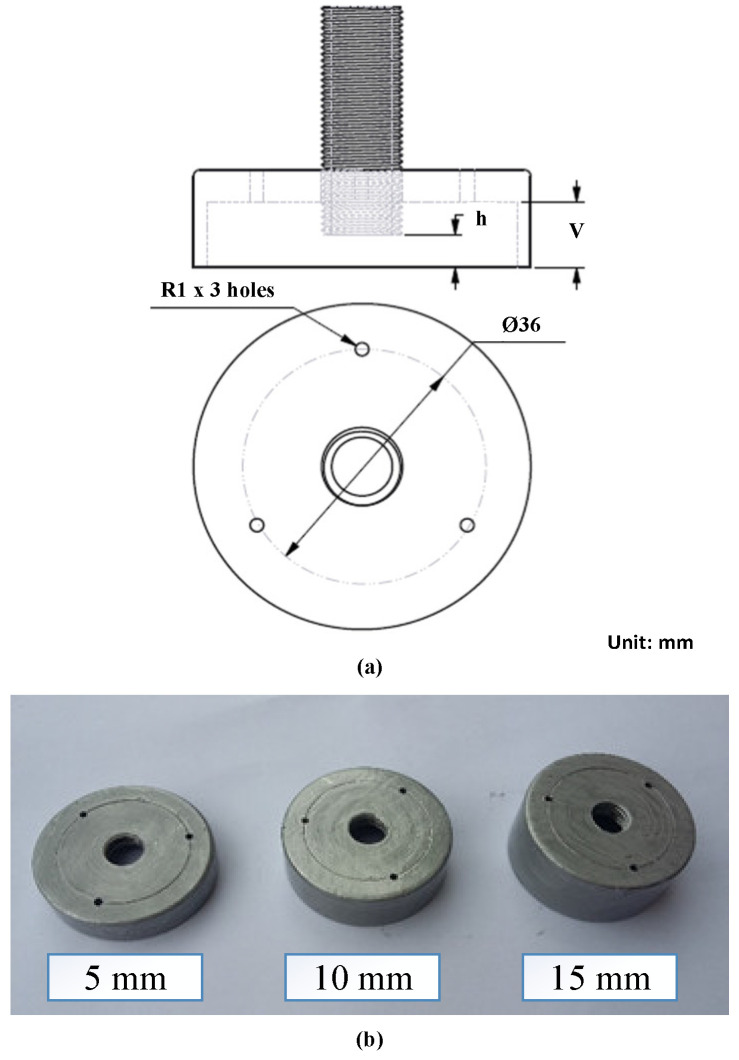
Design (**a**) and manufactured parts (**b**) of the FFD with different air sprue gaps (h) and flow focusing device heights (V).

**Figure 7:**
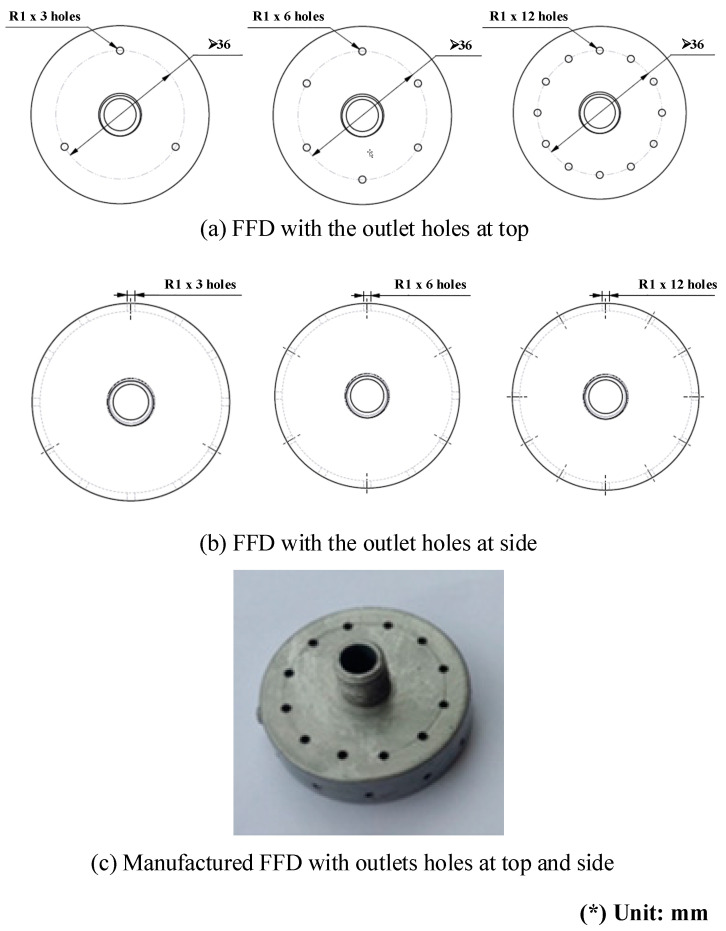
The FFD with outlet holes at the top (**a**) and side (**b**). The manufactured FFD (**c**).

With this structure, to investigate the effect of the air cover on the gas heating process more specifically, many experimental cases were proposed with different FFD types, as shown in Figure 6 and Figure 7. For application in the molding process, the distance between the hot sprue of gas and the cavity surface is an important element, which strongly impacts the heating rate and temperature distribution, especially with the depth cavity. Therefore, in this research, the air sprue gap (h) was studied by changing the h from 5 to 15 mm. In addition, the air focusing volume is also an important element in FFD geometry. With an FFD volume that is too small, the flow of hot air will be reduced. However, with an FFD volume that is too large, the focusing function will not be clear. Therefore, in this paper, the air focusing volume is observed through different FFDs, with the height of the device (V) ranging from 5 to 15 mm. Figure 6 illustrates the experiment conducted to assess the impact of the air sprue gap (h) and the height of the flow focusing device (V) on the efficiency of the gas heating process using many types of air covers with three outlet holes on the top surface. Moreover, for observing the influence of the outlet gate on the heating process, the position and number of outlet holes of FFD were also designed, as shown in Figure 7. This FFD was manufactured with 12 outlet holes at the top and side. Therefore, depending on each case, these outlet holes were opened or closed, in order to observe the influence of the location and number of outlet holes on the heating result.

**Figure 8:**
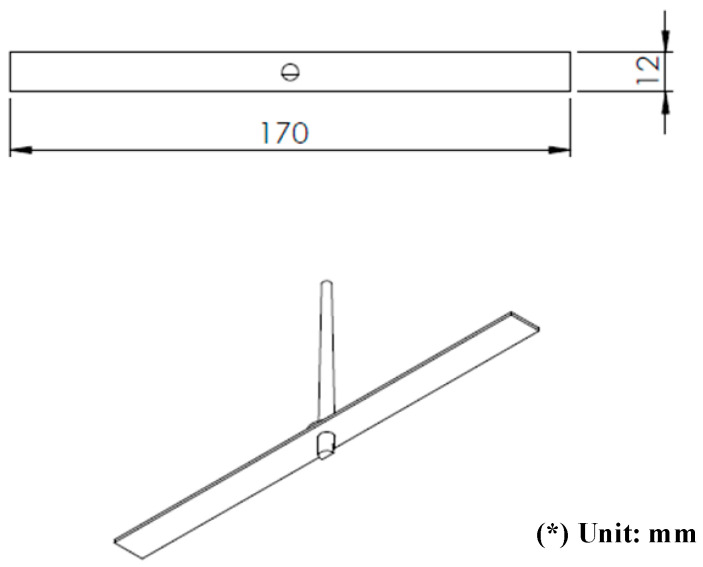
Dimensions of the model used in the flow length experiment.

**Figure 9:**
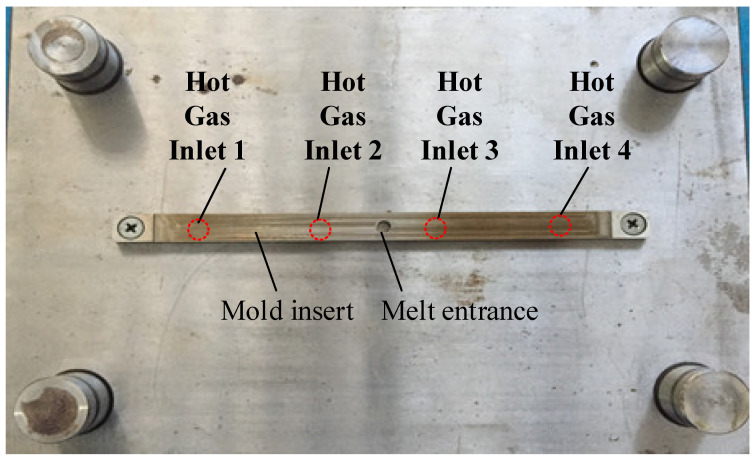
Experimental cavity mold design.

**Figure 10:**
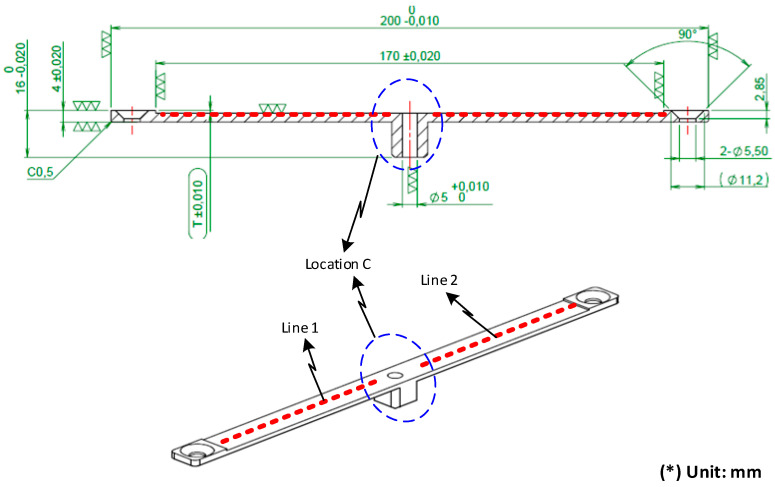
Mold insert design.

After investigating the impact of applying an air cover during the heating process, the air cover was applied to detect the change in the melt flow length when Ex-GMTC was carried out with and without the application of FFD. A thin-walled model, which is presented in Figure 8, was observed to assess any development in the melt flow length. This part had dimensions of 170 mm × 12 mm and a thickness of 0.4 mm. An injection mold with one cavity was designed and manufactured, as shown in Figure 8. In this application, the mold was designed with an insert, as shown in Figure 9. A structure with an insert can help to increase the heating efficiency, while the heating area can also be more easily controlled. Figure 10 shows the dimensions of the insert. The insert has a thickness of 3.6 mm in the heating area. To observe the heating effect, the temperature on lines L1 and L2 was collected and compared. In the heating step, the temperature uniformity of the cavity surface could be increased by the heating of four gates. The element positions of the heating step are shown in Figure 10. Due to the time taken to remove the gas drier to the outside of the molding area, as well as the time taken for the infrared camera to measure the temperature distribution, the experimental results of the temperature distribution were collected at the end of the heating step, with a delay time of 3 s.

**Figure 11:**
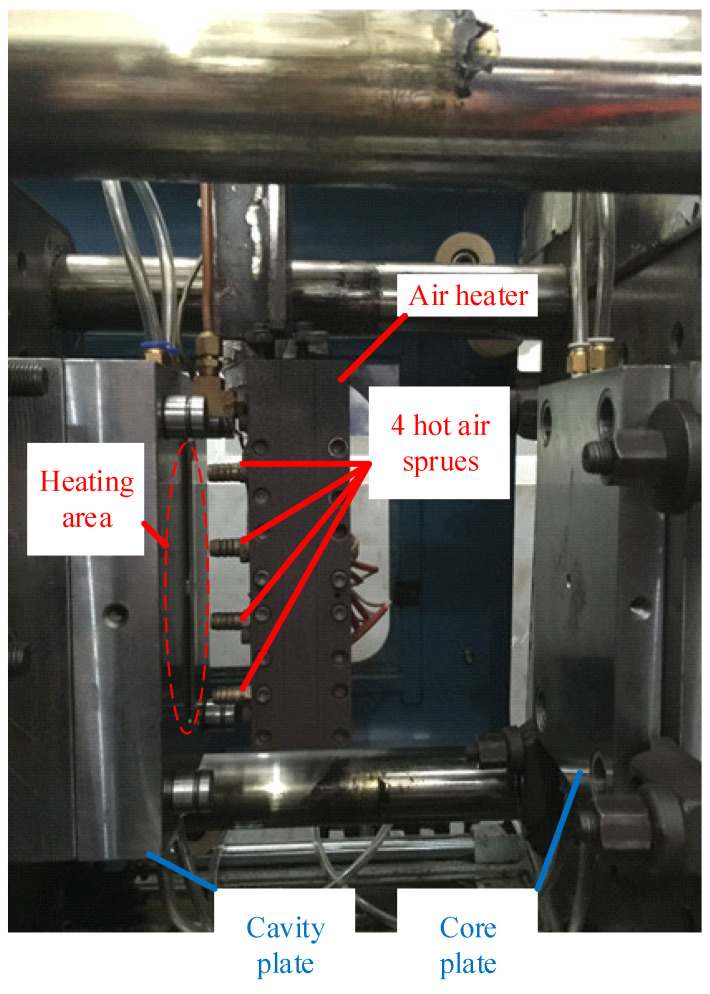
Mold insert in the heating process without the FFD.

**Figure 12:**
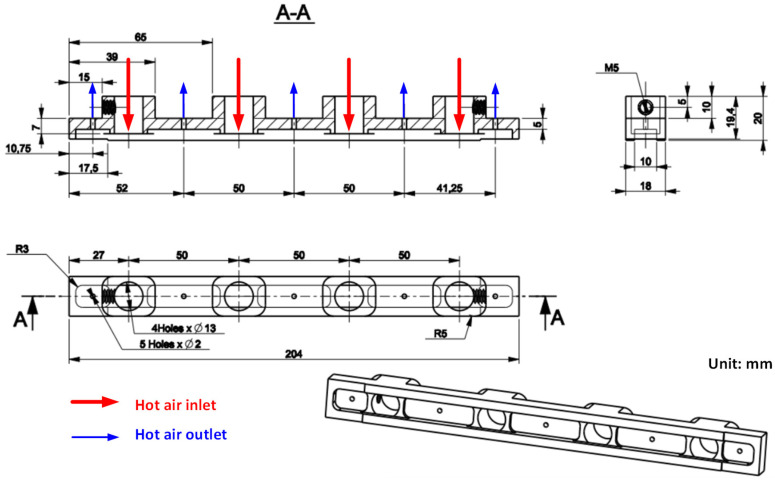
The FFD used for melt flow length research.

**Figure 13:**
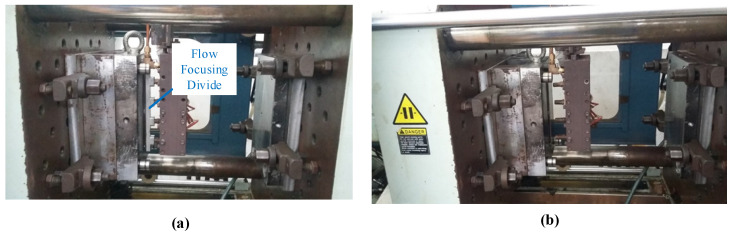
Heating process with (**a**) and without (**b**) the FFD.

To estimate its application to a real mold, Ex-GMTC with the assistance of FFD, as shown in Figure 11 and Figure 12, was applied to increase the heating efficiency of the cavity area before the filling step started. In the experiment, the melt flow length was compared for the cases of “without heating”, and heating “with” and “without” the FFD, as presented in Figure 13. In all cases, the initial temperature of the mold insert was set to 30 °C. For the molding experiment, acrylonitrile butadiene styrene (ABS 750SW from Kumho Petrochemical Company, Seoul, Korea) was used for the molding process, with a filling pressure of 60 kg/cm^2^, filling time of 1.5 s, melt temperature of 200 °C, and cooling time of 20 s. In all experiments, the molds and Ex-GMTC module were assembled by means of an SW-120B molding machine (Shine Well Machinery Co., Ltd., Taichung City, Taiwan) and other equipment, as shown in Figure 2. Figure 13 presents the heating step and the positions of the hot gas generator, hot gas gate, and heating area in the heating step.

### 2.2. Simulation Methods

**Figure 14:**
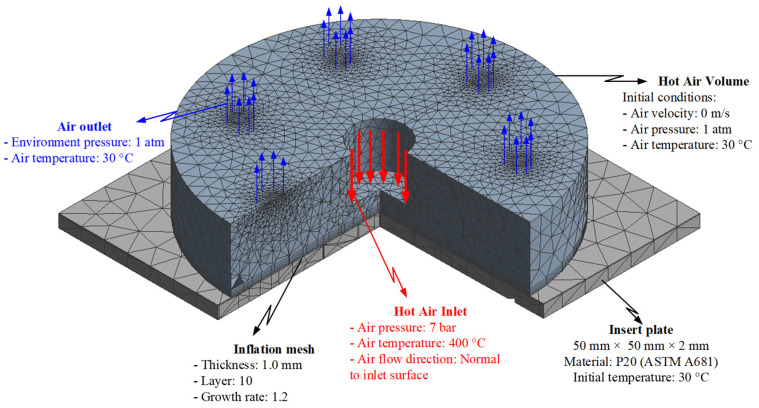
Simulation model of the FFD verification model.

**Figure 15:**
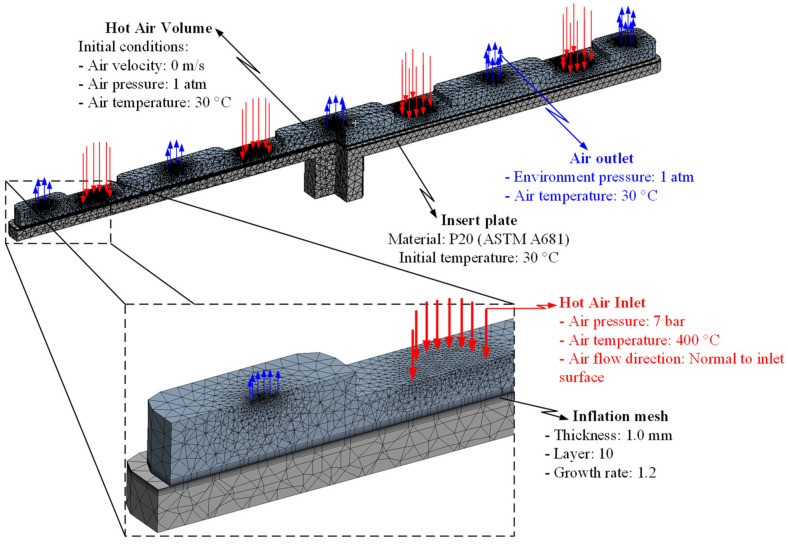
Simulation model of the FFD application model.

For analyzing the temperature distribution of the mold surface, a simulation model was built and run using the ANSYS software (ANSYS, Inc., Hochiminh City, Vietnam). Based on the design of the cavity insert, the simulation models contained two volumes: The insert and hot gas volumes. Figure 14 and Figure 15 show the meshing model and the boundary conditions for simulation, whereas the simulation parameters are presented in Table 1. In the case of the FFD verification model, hot gas was applied at 400 °C under a pressure of 7 bar. The direction of this hot gas flow was set perpendicular to the heating surface. In the simulation, the initial gas volume was set at 30 °C with a pressure of 1 atm. The outlet of the hot gas was set as the opening area, with the environment air at 30 °C and a pressure of 1 atm. In addition, the initial temperature of the insert was set at 30 °C and was comprised of P20 steel. For the FFD application model, the simulation model was operated with four inlets and five outlets of hot gas. In this simulation, the boundary condition was the same as that used in the FFD verification model. In order to improve the simulation precision, a hex-dominant element was used for meshing the insert part. The inflation meshing method was applied with ten layers at the contact surfaces. Moreover, a tetrahedron element was used for the air volume, with a smaller element size applied for the inlet and outlet of the hot air gate. In the simulation, the heat transfer mode around all external surfaces of the mold plate was set to free convection to the air, with an ambient temperature of 30 °C and a heat transfer coefficient of 10 W/m^2^ K. For comparing the influence of the FFD on the heating efficiency, the temperature distribution at the heating surface and the temperature on lines L (Figure 4), L1, and L2 (Figure 10) were collected and compared with the experimental results. For line L, 65 temperature points were collected. For lines L1 and L2, 120 temperature points were collected for a comparison with the experimental results.

## 3. Results and Discussion

### 3.1. Influence of the Air Sprue Gap (h) and Height of the Flow Focusing Device (V) on the Heating Process

Previous studies [22,23,24] have indicated an improvement in the heating effectiveness when external GMTC is operated to increase the mold surface temperature. However, this heating technique is not efficient due to the divergence of the hot gas, as well as the negative influence of the environment. In this paper, for improving the efficiency of Ex-GMTC, the application of an FFD in the mold heating process was suggested, with the heating efficiency being estimated by the temperature distribution on the insert plate. In this step, for heating the insert plate, one hot gas gate was used. The heating step was simulated with the model, as shown in Figure 14, with a gas temperature of 400 °C and a heating time of 20 s. Various types of FFDs were used to find the best shape for the heating process.

**Figure 16:**
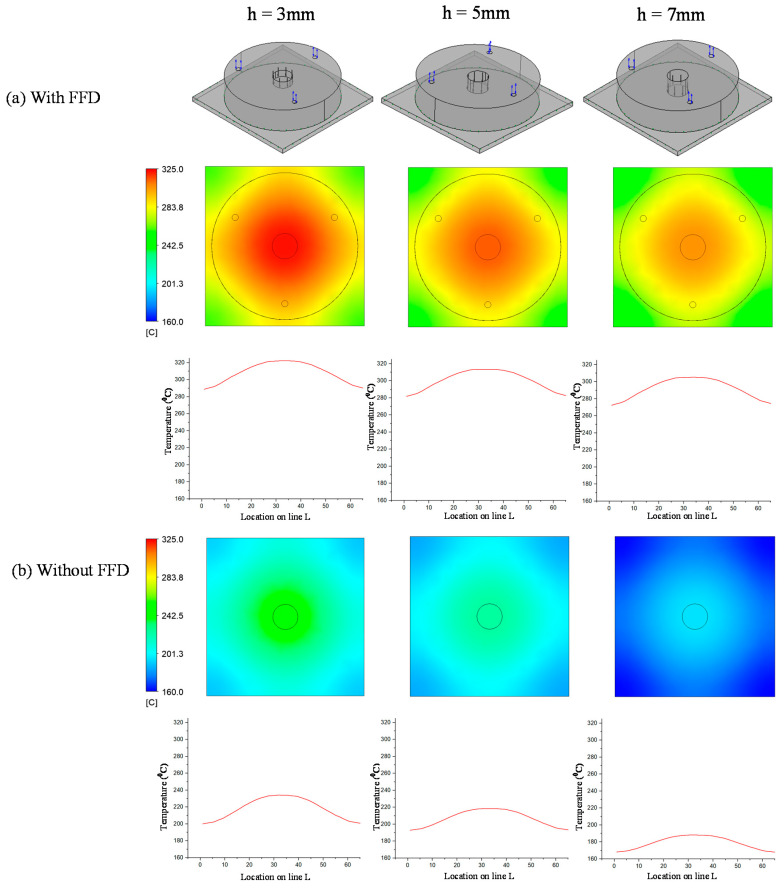
Effects of the air sprue gap h = 3, 5, and 7 (mm) with the height of the FFD V = 10 mm for the temperature distribution with (**a**) and without (**b**) the FFD by simulation.

In previous research, the results showed that the narrower the air sprue gap, the higher the heating rate. However, the temperature distribution was poor. In this research, the temperature distribution on line L (Figure 4) was collected and compared via simulations and experiments. In terms of the simulation, Figure 16 presents the temperature distribution on the insert surface with different air sprue gaps (h) and with and without the FFD. When the FFD was used, the height of the FFD was 10 mm and three outlet holes on top were used. Compared with cases without the FFD, FFD-assisted ex-GMTC proved that the FFD could help to increase the heating rate due the possibility of hot gas being focused at the heating area, which helped to heat the insert surface to over 275 °C. Meanwhile, for cases without the FFD, the heating process could only increase the surface temperature to over 165 °C due to the dispersion of hot air to the environment. With the assistance of the FFD, these results show that the highest temperature was reached with an air sprue gap of 3 mm and a maximum temperature of 322.2 °C; meanwhile, the highest temperature was 305.2 °C with an air sprue gap of 7 mm. With the examination of the temperature difference, the air sprue gap of 7 mm had the best temperature distribution on the insert plate, with the temperature difference being 33.2 °C. In contrast, the 3 mm air sprue gap displayed a slightly higher temperature difference at 35.2 °C. From the simulation results, it can be seen that the air sprue gap greatly affects the temperature and thermal distribution of the insert. The smaller the air sprue gap, the higher the cavity temperature, because a small air sprue gap leads to a greater heat absorption capacity in the insert plate. Moreover, a larger air sprue gap can lead to a better temperature distribution due to hot air moving throughout the cover before escaping.

**Figure 17:**
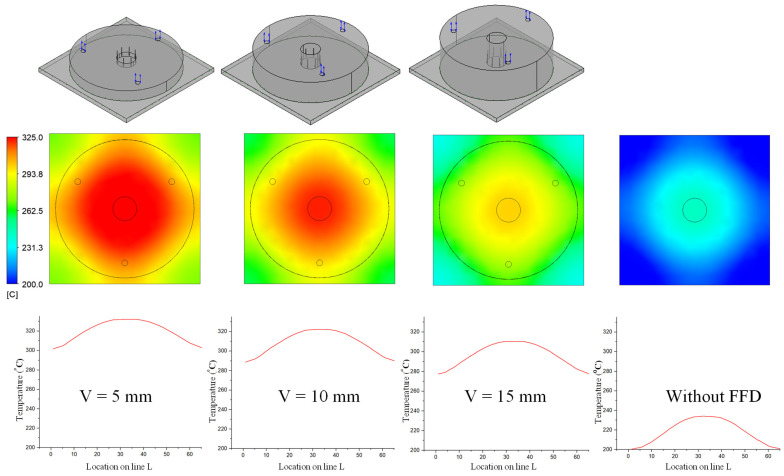
Effects of the height of the FFD V = 5, 10, and 15 mm with the air sprue gap h = 3 mm for the temperature distribution with and without air cover by simulation.

The simulation results given in Figure 17 also illustrate that the height of the FFD (V) also has an effect on the heating rate and temperature distribution. With the height of the FFD varying from 5 to 15 mm, the air sprue gap was 3 mm, and it can be seen that, with the case of the 5 mm FFD height, the temperature reached the maximum value of 332.2 °C, but had the worst temperature distribution. In contrast, in the case of V = 15 mm, although the center temperature was only 310.4 °C, the temperature difference was reduced. Therefore, the results show that the height of the FFD has a significant effect on the temperature and thermal distribution of the insert. The smaller the height of the FFD, the higher the cavity temperature, because FFDs with a smaller height lead to a better heat absorption capacity within the insert plate. In contrast, FFDs with larger heights can allow for a better temperature distribution because of the hot air condensation that occurs in the cover before the air escapes, meaning that the hot air will transmit heat to lower temperature regions.

**Figure 18:**
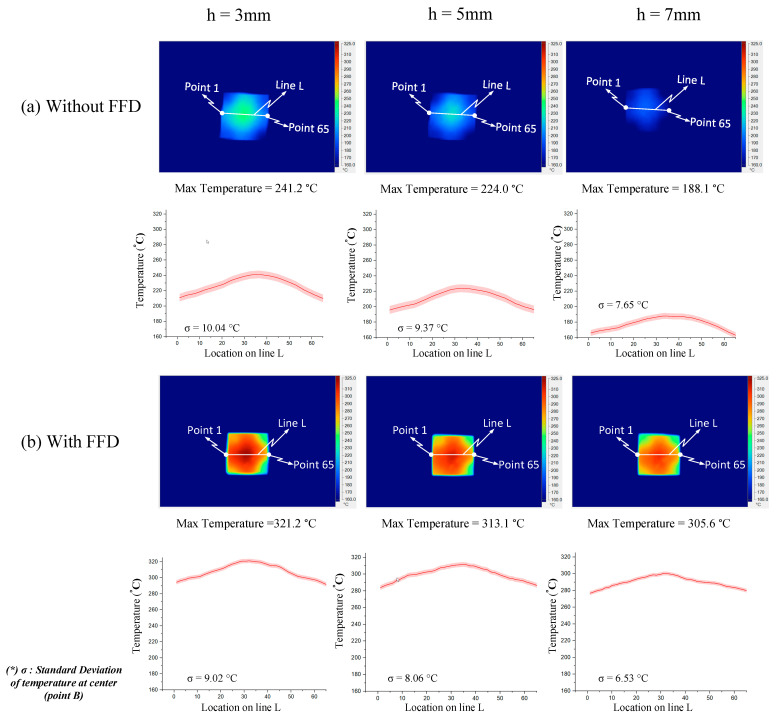
Effects of the air sprue gap h = 3, 5, and 7 mm with the height of the FFD V = 10 mm for the temperature distribution with and without an air cover in the experiment.

**Figure 19:**
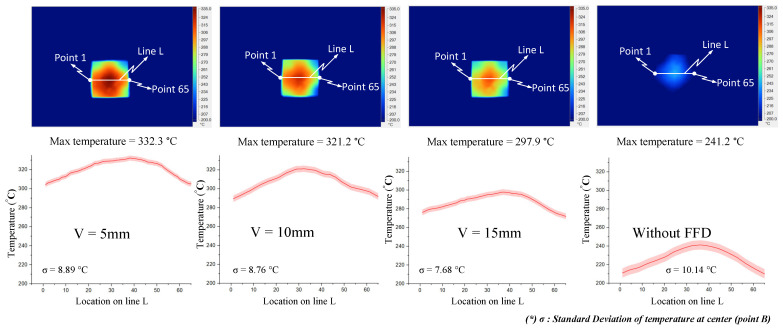
Effects of the height of the FFD V = 5, 10, and 15 mm with the air sprue gap h = 3 mm for the temperature distribution with and without an air cover in the experiment.

**Figure 20:**
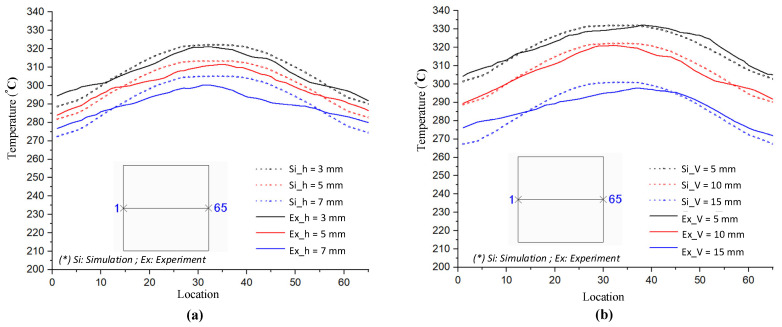
Temperature distribution comparison of the simulation and experiment with various air sprue gaps (**a**) and heights of the flow focusing device (**b**).

In order to validate the simulation results, an experiment was carried out with the same simulation boundary conditions. The surface temperatures at the insert plate were measured by an infrared thermal camera and compared with the simulation results. Figure 18 shows that the air sprue gap significantly affects the temperature of the insert and the air sprue gap of 3 mm gives the best results (321.2 °C). Likewise, in Figure 19, the actual results show that the volume of air actually influences the temperature of the insert plate and an FFD height of 5 mm gives the best results (332.3 °C). In both cases, the actual thermal diagram has the same parabolic shape as the simulation result. In this study, the temperature on line L was collected 10 times, and the standard deviation (σ) was calculated and present as in Figure 18 and Figure 19. In the experiment, the temperature standard deviation varies from 6.53 to 10.14 °C. For comparing the simulation and experiment results, the temperature on line L was collected and compared, as shown in Figure 20. This comparison shows that the temperature profiles of the simulation and experiment exhibit good agreement, with the highest temperature at the center and the lowest temperature at the side of the insert. In all cases, the highest temperature of the experimental cases is slightly lower than the experimental cases; meanwhile, the simulation temperatures at the side are lower in the simulation than in the experimental cases. Comparing the temperature distribution on line L, these results show that the maximum temperature was reached with an air sprue gap of 3 mm. The difference in temperature follows line L in cases from 25 to 32 °C and, in the case of the air sprue gap, 3 mm is the highest. The temperature at the end of the heating step, in the case of the FFD, is always higher than in the case without the FFD, by about 50–70 °C. In addition, the height of the FFD was also investigated. With a height of 5 mm, the insert temperature reached the highest values. The difference in temperature in these cases is about 10–15 °C; the max-min temperature difference is about 30–40 °C; and, in the case of an FFD height of 5 mm, the temperature when using a cover is always higher than without a cover, by about 70–90 °C. The heat transfer from the insert center to the side is the main reason for this phenomenon. In the heating process, the insert center absorbs the thermal energy more easily than in other areas due to closing to the hot sprue air; thus, at the end of the heating process, the temperature at the center is the highest. When the heating process is finished, the heat support to the insert will be stopped, so heat is only transferred from the higher temperature to lower temperature, meaning that the temperature at the center is decreased and the temperature at the side is increased due to the heat transfer from the center. Therefore, as displayed in Figure 20, the simulation profile shows the temperature at the end of the heating step. However, in the experiment, there is a delay in the time taken for the thermal camera to capture the temperature value; thus, heat transfer occurred and led to a decreasing temperature at the center and increasing temperature at the side. Figure 20 also shows that the temperature uniformity is improved after the heating step is finished due to the heat transfer from the higher temperature location to the lower temperature area, which is an advantage for the application of this method in the injection molding process, which needs about 3 s to remove the heating equipment and for the mold to close, as shown in steps 4 and 5 of Figure 1.

### 3.2. Influence of the Outlet Gates on the Heating Process

**Figure 21:**
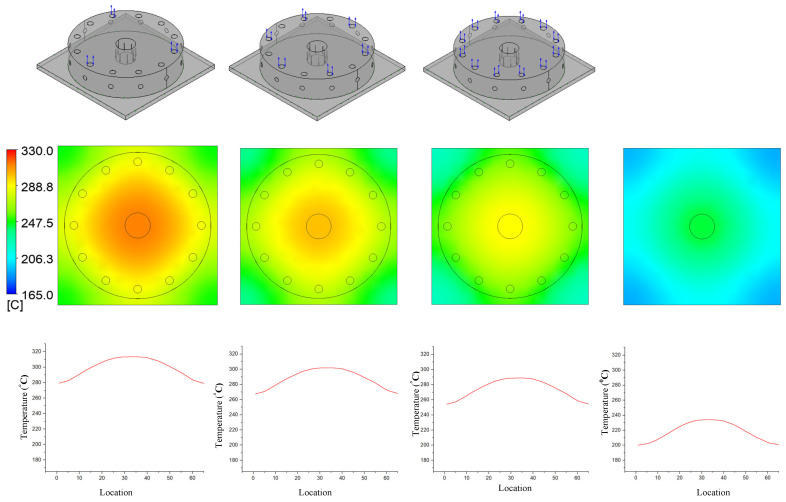
Effects of outlet holes on the top with the air sprue gap h = 3 mm and height of the FFD V = 10 mm for the temperature distribution with and without the air cover in the simulation.

**Figure 22:**
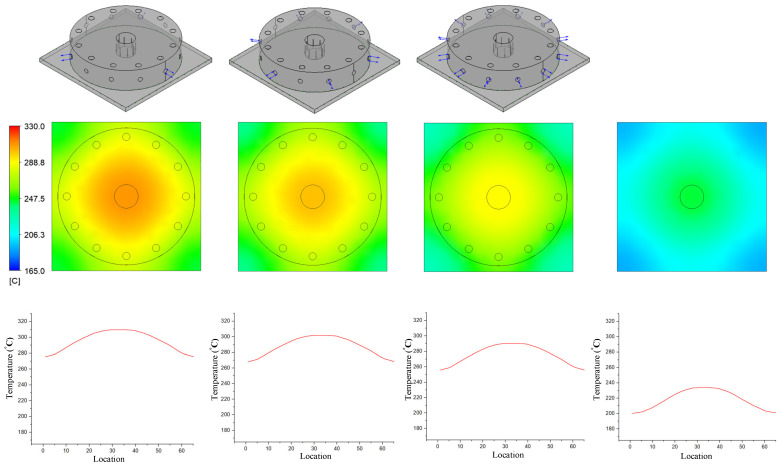
Effects of outlet holes on the side with the air sprue gap h = 3 mm and height of the FFD V = 10 mm for the temperature distribution with and without the air cover in the simulation.

Figure 21 and Figure 22 indicate the effect of the number of outlet holes in the air cover and its position on the heating rate and temperature distribution by simulation. The simulation was conducted with h = 3 mm and V = 10 mm, and 3, 6, and 12 holes on both the top and side surfaces were tested. The results show that, in both cases, a smaller number of outlet holes leads to a higher insert plate temperature because, in this case, the air will hardly be able to escape, leading to a higher air density and more heat energy, resulting in a higher insert temperature. In contrast, the more outlet holes there are, the more uniform the heat distribution is, but the temperature drops very quickly and the air flow velocity also decreases. Specifically, the highest temperatures were reached with three outlet holes on the top surface and side surface. These temperatures were 313.5 and 309.6 °C, respectively, but resulted in a poor temperature distribution. On the other hand, an air cover with 12 outlet holes on the top and side surface provided the lowest temperature (288.9 and 290.3 °C, respectively); however, this air cover had the best temperature distribution. It can be noted that outlet holes on the top surface result in a higher temperature on the side surface because the distance traveled to the top surface is longer than to the side surface, while the higher air density leads to a higher insert plate temperature. Moreover, air escapes at the top surface more easily than it escapes on the side surface, so new air is continuously exchanged, resulting in higher insert plate temperatures. Therefore, the number of air holes has the greatest effect on the temperature and thermal uniformity of the insert, and we should choose as small values as possible to increase the temperature and distribute the air holes properly.

The results of the temperature for line L with various numbers of outlet holes on the top and side surface of the FFD also show that, with three outlet holes, the insert plate reached the highest temperature and reached the lowest temperature when using the air cover with 12 outlet holes. Moreover, the difference in temperature was about 20 °C and the max-min difference in temperature was about 34 °C, and it can be noted that the temperature when using the air cover is always higher than without the FFD, leading to a 50−80 °C temperature difference.

**Figure 23:**
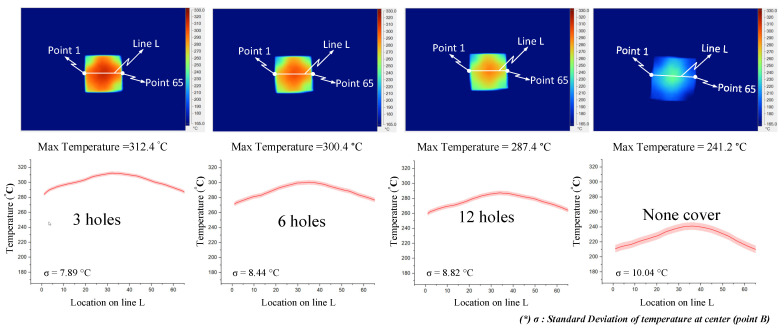
Effects of outlet holes on the top with the air sprue gap h = 3 mm and height of the FFD V = 10 mm for the temperature distribution with and without the air cover in the experiment.

**Figure 24:**
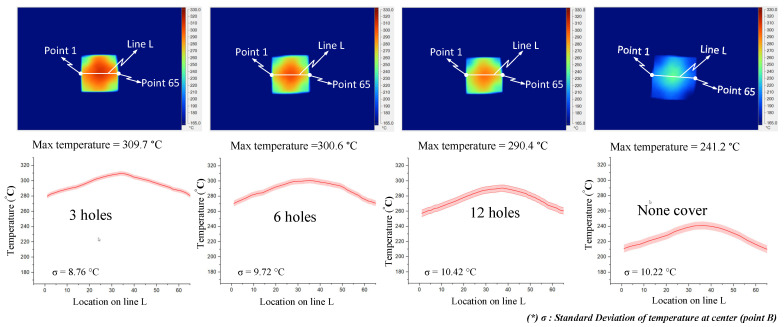
Effects of outlet holes on the side with the air sprue gap h = 3 mm and height of the FFD V = 10 mm for the temperature distribution with and without the air cover in the experiment.

**Figure 25:**
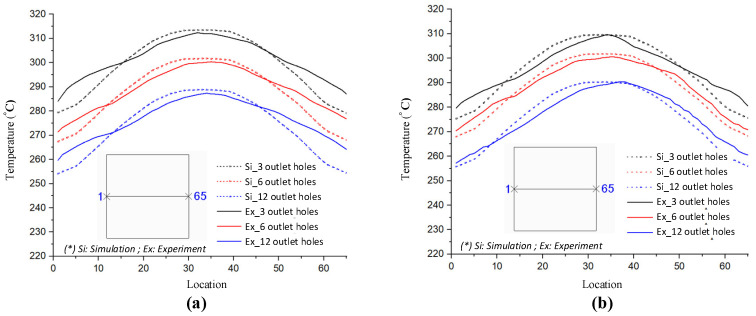
Temperature distribution comparison with various outlet gates on the top (**a**) and side (**b**) of the FFD.

As demonstrated in Figure 23 and Figure 24, the number of outlet holes and the position of the air cover were also examined by an experiment. The actual results show that the air outlet holes actually affect the temperature of the insert. In both cases, the fewer outlet holes there are, the higher the insert plate temperature, and the air cover with three outlet holes gives the best results, with maximum temperatures of 312.4 and 309.7 °C for outlet holes on the top and side surfaces, respectively. In addition, the actual thermal diagram has the same profile as the simulation result. Moreover, the actual results show that the distribution of the upper outlet holes will lead to a higher insert temperature. In our assessment of various numbers of outlet holes on the top surface, the maximum temperature was reached with three outlet holes, and the difference in temperature for line L varied from 10 to 20 °C, while the max-min temperature difference of the heating surface was about 28 °C. Without the FFD, it was about 31 °C. In addition, with the same heating time, the temperature when using the FFD was always higher than without the air cover (by about 50–80 °C). In our assessment of various numbers of outlet holes on the top surface, the maximum temperature was reached with three outlet holes, while the difference in temperature ranged from about 15 to 30 °C, and the max-min temperature difference ranged from about 28 to 37 °C. With the same heating time, the temperature when using the air cover was always higher than without the cover, by about 40–70 °C. With different outlet hole locations and different numbers of holes, it is clear that using the FFD in the heating process can increase the mold temperature more effectively than the heating process without the air cover. In this research, the temperature on line L was collected 10 times, and the standard deviation (σ) was calculated and is presented in Figure 23 and Figure 24. In the experiment, the temperature standard deviation varied from 7.89 to 10.42 °C. After investigating the simulation and experimental results, a comparison was conducted, as shown in Figure 25. In all cases, it can be seen that the thermal lines representing the results of the experiments and the simulations are quite close together. The same is true for the air sprue gap (h) and the height of the flow focusing device (V), but the heat transfer from the higher temperature area to the lower temperature area causes the max-min temperature difference of the experiment to be slightly smaller than the simulation results, so the actual temperature distribution on the insert is more uniform than the thermal distribution on the simulation. However, although there is a small difference in the distribution of heat, the simulation results basically show the same results as the experiment.

### 3.3. Apply the Ex-GMTC with the Assistance of the FFD in the Mold Heating Step for Increasing the Melt Flow Length in Thin Wall Injection Molding

**Figure 26:**
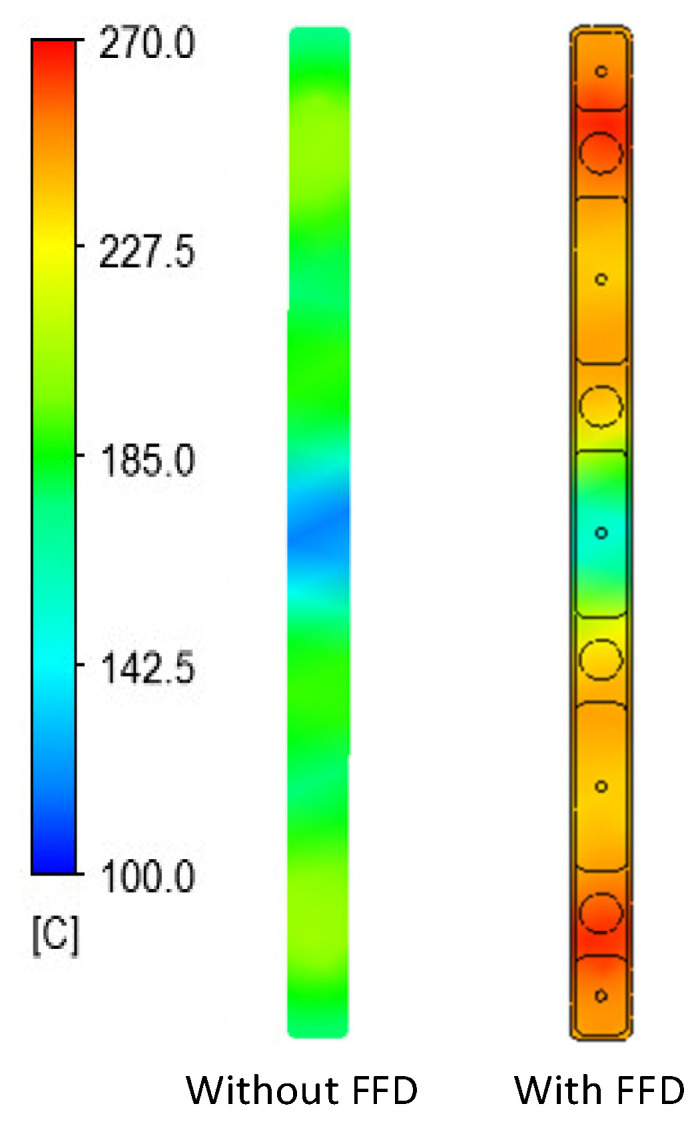
Temperature distribution of the mold insert in the heating process with and without the cover in the simulation.

**Figure 27:**
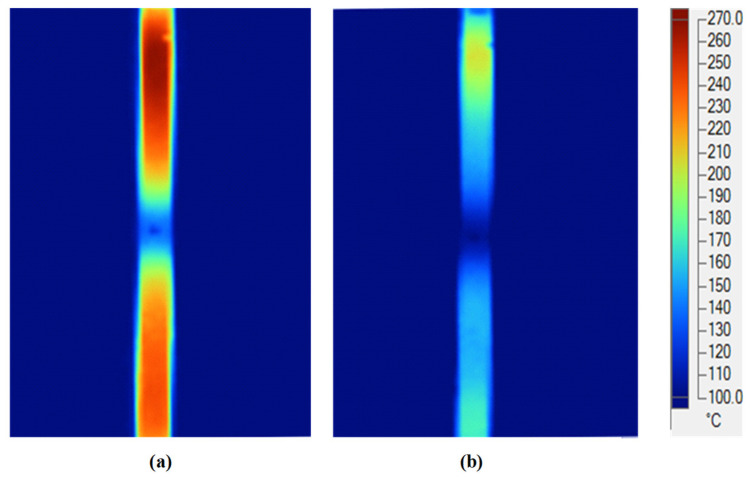
Temperature distribution of the mold insert in the heating process with (**a**) and without (**b**) the FFD in the experiment.

After assessing the advantages of applying an air cover in the heating process based on the results described above, an FFD for melt flow length research was designed, as shown in Figure 12, for application in the injection molding process of a long, thin-walled product, in order to observe its effect on the melt flow length. Figure 26 shows the simulation results for temperature. It is clear that, when using the FFD, the temperature is higher than when no cover is used, by about 70 °C. The simulation results show that the temperature difference between line L1 and line L2 in both cases (with and without the FFD) is not clear and the temperature lines tend to decrease in the middle of the insert plate. To verify the simulation results, an experiment was conducted, and the results of the temperature distribution are shown in Figure 27. In general, the maximum temperature when using the FFD is about 50 °C higher than in the case without the air cover. On the contrary, with the simulation, the difference between the temperature of lines L1 and L2 in the experimental cases with and without the FFD are quite clear, especially without the FFD. In addition, both the simulation and experimental results show that the higher temperatures tend to appear at the two sides of the insert. In conclusion, through the results of the experiments, it can be noted that the application of FFD leads to a significant increase in the mold cavity surface compared to heating without an air cover.

**Figure 28:**
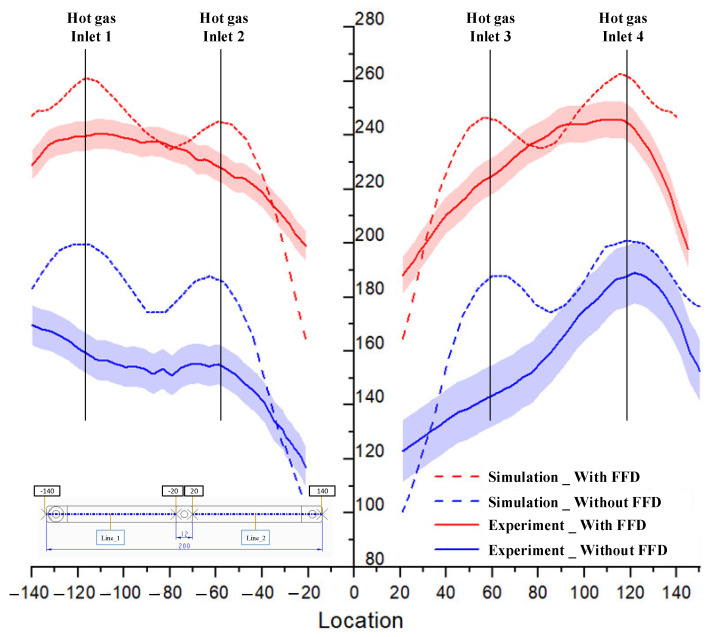
Temperature distribution on the center line of the insert with and without the FFD.

To compare the simulation and experiment in more detail, the temperatures on lines L1 and L2 (Figure 10) were collected and compared, as shown in Figure 28. According to this result, the simulation results show that, at the end of the heating time, the influence of the hot gas inlet locations is very clear, and the result is a higher temperature at these locations. This simulation result appeared in cases with and without the FFD. The reason for this is that the closer the hot gas inlet is, the higher the thermal energy supported will be, meaning that these locations of the insert will rapidly acquire more thermal energy than other areas. As a result, the insert surface reaches a higher temperature close to the inlet of hot gas. In addition, both the simulation and experiment show that the temperature at the center of the mold insert is clearly lower than that in other areas. This result is due to the structure of the insert, which has more material in the center area (Location C in Figure 10), meaning that more energy is required to increase the temperature in this location. In other words, location C can absorb the thermal energy from other areas, and also from hot air. As a result, areas close to location C will easily lose thermal energy, resulting in a lower temperature.

Compared with the experimental results, the temperature profiles also change. With the simulation, the highest temperature also appears at hot gas inlets 1 and 4. However, the temperature will decrease continuously in the center of the side of the mold insert. Moreover, the higher temperature found at the hot gas inlets 2 and 3 has almost completely disappeared. This result is due to the fact that, during the time delay used to measure the temperature distribution, which is about 3 s, the thermal energy from a higher temperature location was transferred to a lower temperature location, which is location C (Figure 10). As a result, the temperature cap at hot gas inlets 2 and 4 was removed. In addition, the experimental temperatures close to location C tend to be higher than the simulation because of the thermal energy received from the higher temperature area. This phenomenon appeared with or without the FFD.

In the experiment, the temperature profiles of lines L1 and L2 were also different with and without the FFD. When the FFD is applied, the temperature balance between L1 and L2 is quite good. However, without the FFD, the temperature of L1 is clearly lower than that of L2. As found in other research, this phenomenon is due to the influence of air flow from the environment, which impacts the flow of hot air. In other words, without the FFD, the hot air flow was interfered with, so the heating effect was unstable. With the FFD, the stability of the heating step is proved by the balance of the temperature profiles between L1 and L2.

In general, the FFD assisted ex-GMTC, which reduced the imbalance in heating between the L1 and L2 sides and improved the heating rate. With the design of the mold insert, as shown in Figure 10, a lower temperature will occur at the center; however, this type of temperature distribution (Figure 26 and Figure 28) is good for assisting with the melt flow, which requires a higher temperature in the area furthest from the melt entrance (Figure 9). The temperature on line L1 and L2 was collected 10 times under the cases of with and without the FFD, and the standard deviation (σ) was calculated and is presented in Figure 28. This result shows that the temperature standard deviation varies from 12.4 to 20.05 °C. In general, the standard deviation of the temperature in the case without the FFD is always higher than in the case with the FFD. This means that the heating step with the FFD assistance is more stable than the case of without the FFD.

For estimating the influence of Ex-GMTC with the assistance of FFD on the filling step, the heating process was achieved with a 400 °C gas temperature and a 20 s heating time. When the heating step finished, the gas drier was removed from the molding area and the filling step was started by completely closing the two-half mold plates. In this stage, the temperature distribution of the cavity surface will change, and the temperature distribution at the end of this step impacts the filling of the melt. In a real molding cycle, it takes about 3 s from the end of the heating period to the start of the filling step. In each case, the molding cycle was operated until the system was stable; then, 10 molding samples were collected for measuring the melt flow length. These results were used to compare the influence of the heating step.

**Figure 29:**
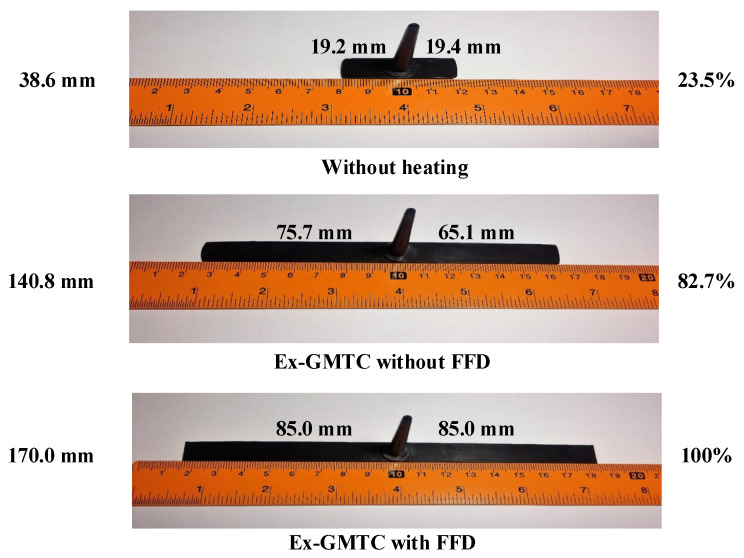
Product flow length comparison.

**Figure 30:**
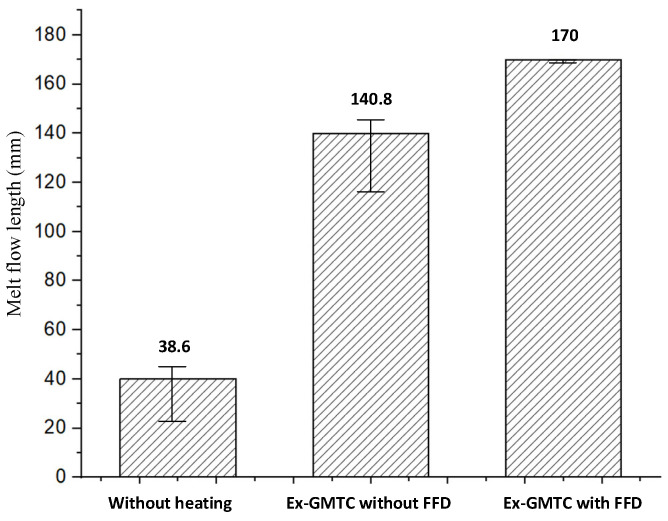
The improvement in the melt flow length.

In the experiment, the injection molding process was run with the application of an FFD. The molding parts were measured, and the results are shown in Figure 29. With the same molding conditions, the experiment was performed for three cases: A traditional molding cycle (without heating); heating by Ex-GMTC without an FFD; and heating by Ex-GMTC with the assistance of an FFD. The results show that the application of an FFD in Ex-GMTC has a positive influence on the filling of the hot melt. Without heating, the melt flow length only reached 23.5% of its fill capacity, with a total length of 38.6 mm; however, with the application of Ex-GMTC, the melt flow length could be increased to 82.7% (L = 140.8 mm). However, in this case, an imbalance in the melt flow was observed with a length difference between the left and right, which measured 75.7 and 65.1 mm, respectively. This imbalance in filling is due to the difference in the insert temperature, which was mentioned above. Next, when applying the FFD for Ex-GMTC, the melt flow length reached its full capacity at 100% (L = 170 mm). For this experiment, the improvement in flow length is presented in Figure 30. The improvement in filling is caused by the reduction in the frozen layer of the melt flow, which allowed the melt to flow much more easily in the filling step of the molding cycle. The standard deviation was calculated for these cases. The results show that without the FFD assistance, the standard deviation of the melt flow length tends to be higher than in the traditional case. This is due to the fact that heating without the FFD let the temperature difference at the cavity surface become larger, so the stability of the filling step was reduced. In the case of FFD-assisted Ex-GMTC, because the cavity was full, the standard deviation was close to zero.

## 4. Conclusions

In this research, simulations and experiments were conducted in order to estimate the temperature distribution when using an air cover in the heating process and its application in terms of improving the melt flow length in the filling step of the injection molding cycle. The most important conclusions drawn can be simplified as follows:

- The air sprue gaps, height of the FFD, location of the hot air outlet, and number of hot air outlets are the main elements that have an effect on the heating process. Through experiments and simulations, the best heating process was found to be the one with the smallest air sprue gaps and lowest FFD. The three hot air outlet holes at the top of the FFD show a better heating efficiency than the side outlet;
- The heating rate was clearly improved by applying an FFD for the assistance of Ex-GMTC due to its ability to focus the hot air to the heating location;
- After finished the heating process, the temperature distribution of the insert is changed due to the heat transfer from the higher temperature to lower temperature area. This is a positive effect because it improves the temperature uniformity;
- In the application case, the melt flow length model was used to test the ability of Ex-GMTC with the assistance of an FFD. The results show that the insert thickness has a strong impact on the temperature distribution, as well as the change in the temperature profile after the heating step. In this research, the heating step was carried out for 20 s, and the cavity surface was heated to over 180 °C, which was high enough to reduce the negative effects of the frozen layers found in almost all common plastic materials. From the melt flow length results, we can see that, in the case without the FFD, an imbalance in the melt flow was observed, with a difference between the left and right sides of about 10.6 mm. Ex-GMTC with the FFD was proven to provide a stable heating step, which helped to improve the temperature balance between the two sides of the cavity and the result is an improved melt flow balance. For the molding cycle test, the heating process with the FFD helped to raise the mold temperature, allowing the melt to completely fill the cavity.

## Figures and Tables

**Table 1:** Parameters employed for the Ex-GMTC simulation.

Parameters	Unit	Value
Hot air temperature	°C	400
Density of hot air [25]	kg/m^3^	0.524
Heat capacity of hot air [25]	J/kg·K	1068
Thermal expansion coefficient of hot air [25]	×10^−3^/K	1.52
Density of steel P20 (ASTM A681)	kg/m^3^	7861
Heat capacity of steel P20 (ASTM A681)	kJ/kg·K	1926
Thermal conductivity of steel P20 (ASTM A681)	W/m·K	41.5
Simulation type	-	Transient

## Data Availability

The data used to support the findings of this studyare available from the corresponding author upon request.

## References

[1] Chen S.C., Chien R.D., Lin S.H., Lin M.C., Chang J.A. (2009). Feasibility Evaluation of Gas-Assisted Heating for Mold Surface Temperature Control during Injection Molding Process. Int. Commun. Heat Mass.

[2] Yang Q., Guo W., Meng Z., Mao H., Hua L., Liu Y. (2020). Investigation on Forming Defects and Crystallization of Plastic Parts in Combined in-Mold Decoration and Microcellular Injec-Tion Molding Based on a Multiphase Flow-Solid Coupled Heat Transfer Model. Int. J. Heat Mass Transf..

[3] Macedo C., Freitas C., Brito A.M., Santos G., Faria L., Laranjeira J., Simoes R. (2019). Influence of Dynamic Temperature Control on the Injection Molding Process of Plastic Components. Procedia Manuf..

[4] Jeng M.C., Chen S.C., Minh P.S., Chang J.A., Chung C. (2010). Rapid Mold Temperature Control in Injection Molding by Using Steam Heating. Int. Commun. Heat Mass Transf..

[5] Guilong W., Guoqun Z., Huiping L., Yanjin G. (2010). Analysis of Thermal Cycling Efficiency and Optimal Design of Heating/Cooling Systems for Rapid Heat Cycle Injection Molding Process. Mater. Des..

[6] Hammami M., Kria F., Baccar M. (2015). Numerical Study of Thermal Control System for Rapid Heat Cycle Injection Molding Process. J. Process. Mech. Eng..

[7] Li J., Li T., Peng X., Liu F., Zhou H., Jiang S. (2018). Optimal Design of Heating System for Electrical Rapid Heat Cycle Mold Based on Multi-Objective Optimization, Multiple-Attribute Decision-Making, and Conformal Design Theory. Adv. Mech. Eng..

[8] Sánchez R., Martinez A., Mercado D., Carbonel A., Aisa J. (2021). Rapid heating injection moulding: An experimental surface temperature study. Polym. Test..

[9] Chang P.C., Hwang S.J. (2006). Experimental Investigation of Infrared Rapid Surface Heating for Injection Molding. J. Appl. Polym. Sci..

[10] Chang P.C., Hwang S.J. (2006). Simulation of Infrared Rapid Surface Heating for Injection Molding. Int. J. Heat Mass Transf..

[11] Yu M.C., Young W.B., Hsu P.M. (2007). Micro-Injection Molding with the Infrared Assisted Mold Heating System. Mater. Sci. Eng. A.

[12] Hopmann C., Weber M., Schöngart M., Schäfer C., Bobzin K., Bagcivan N., Brögelmann T., Theiß S., Münstermann T., Steger M. (2015). Injection Moulding of Optical Functional Micro Structures Using Laser Structured, Pvd-Coated Mould Inserts. AIP Conf. Proc..

[13] Evens T., Malek O., Castagne S., Seveno D., Van Bael A. (2020). A Novel Method for Producing Solid Polymer Microneedles Using Laser Ablated Moulds in an Injection Moulding Process. Manuf. Lett..

[14] Chen S.C., Jong W.R., Chang Y.J., Chang J.A., Cin J.C. (2006). Rapid Mold Temperature Variation for Assisting the Micro Injection of High Aspect Ratio Micro-Feature Parts Using Induction Heating Technology. J. Micromech. Microeng..

[15] Yao D., Kimerling T.E., Kim B. (2006). High-Frequency Proximity Heating for Injection Molding Applications. Polym. Eng. Sci..

[16] Chen S.C., Minh P.S., Chang J.A., Huang S.W., Huang C.H. (2012). Mold Temperature Control Using High-Frequency Proximity Effect Induced Heating. Int. Commun. Heat Mass Transf..

[17] Lin H.L., Chen S.C., Jeng M.C., Minh P.S., Chang J.A., Hwang J.R. (2012). Induction Heating with the Ring Effect for Injection Molding Plates. Int. Commun. Heat Mass Transf..

[18] Guerrier P., Tosello G., Nielsen K.K., Hattel J.H. (2015). Three-Dimensional Numerical Modeling of an Induction Heated Injection Molding Tool with Flow Visualization. Int. J. Adv. Manuf. Technol..

[19] Chen S.C., Minh P.S., Chang J.A. (2011). Gas-Assisted Mold Temperature Control for Improving the Quality of Injection Molded Parts with Fiber Additives. Int. Commun. Heat Mass Transf..

[20] Chen S.C., Chang J.A., Hsu W.Y., Huang S.W. (2011). Improvement of Replication Accuracy of Micro-Featured Molding Using Gas-Assisted Heating for Mold Surface. Microelectron. Eng..

[21] Chen S.C., Lin C.Y., Chang J.A., Minh P.S. (2013). Gas-Assisted Heating Technology for High Aspect Ratio Microstructure Injection molding. Adv. Mech. Eng..

[22] Minh P.S., Trung D.T., Uyen T.M.T. (2018). The Feasibility of External Gas-Assisted Mold-Temperature Control for Thin-Wall Injection Molding. Adv. Mech. Eng..

[23] Tran M.T.U., Nguyen T.G., Trung D.T., Tran A.S., Minh P.S. (2020). External Gas-Assisted Mold Temperature Control Improves Weld Line Quality in the Injection Molding Process. Materials.

[24] Phan T.N., Thanh T.D., Tran A.S., Pham S.M. (2019). Study on External Gas-Assisted Mold Temperature Control for Improving the Melt Flow Length of Thin Rib Products in the Injection Molding Process. Adv. Polymer Technol..

[25] Baehr H.D., Stephan K. (2006). Heat and Mass Transfer.

